# Effects of MP-AzeFlu enhanced by activation of bitter taste receptor TAS2R

**DOI:** 10.1186/s13223-020-00438-w

**Published:** 2020-06-03

**Authors:** Sandra Ekstedt, Susanna Kumlien Georén, Lars Olaf Cardell

**Affiliations:** 1 Division of ENT Diseases, Department of Clinical Science, Intervention and Technology, Karolinska Institutet, Stockholm, Sweden; 2 Department of ENT Diseases, Karolinska University Hospital, Stockholm, Sweden

**Keywords:** Azelastin, Fluticasone, Bitter taste, TAS2R, Allergic rhinitis, Allergy

## Abstract

MP-AzeFlu is relatively new a pharmaceutical drug used in the treatment of allergic rhinitis. It is comprised of azelastine hydrochloride (AZE), a potent histamine-H1-receptor antagonist and fluticasone propionate (FP), corticosteroid. It’s somewhat bitter taste (often considered a disadvantage) can be attributed to AZE. We here hypothesize that MP-AzeFlu may induce some of its beneficial effects through activation of bitter taste receptors (Tas2R), which have recently been described in human airways. In the nose Tas2Rs induce secretion of antimicrobial peptides and increase ciliary activity, while in the lung they cause airway smooth muscle relaxation. The mechanisms behind Tas2R-mediated effects are not yet fully known. In order to evaluate the role of Tas2R in the effects induced by MP-AzeFlu the dilatory response of pre-contracted isolated airways from Balb/c mice was investigated in tissue bath myographs in the presence or absence of various well-characterized pharmacological antagonists or their corresponding vehicles. MP-AzeFlu caused a potent dose-dependent relaxation of pre-contracted airways, an effect probably mediated by its AZE component. The dilatory effect of MP-AzeFlu and AZE both mimicked the response induced by the Tas2R agonist, chloroquine, but was independent of histamine receptor (H1-, H2- and H3-), prostaglandins, cAMP and cGMP involvement, all known to be common pathways for airway dilation. Other bitter-tasting antihistamines (i.e. olopatadine and desloratadine) also relaxed airway segments. These data support the notion that MP-AzeFlu has the ability to activate Tas2R in the same way as chloroquine. The effect appears to be mediated by AZE, but not via the histamine receptor. Activation of Tas2R by MP-AzeFlu may contribute to its superior efficacy over FP observed in controlled clinical trials in patients with moderate/severe allergic rhinitis.

## To the editor

MP-AzeFlu (Dymista^®^, Meda, Solna, Sweden) is a new class of allergic rhinitis (AR) treatment, comprising an intranasal anti-histamine (azelastine hydrochloride [AZE]), and an intranasal corticosteroid (INS) (fluticasone propionate [FP]). Its efficacy as a first line therapy, has been documented in randomized controlled trials (RCTs) [1, 2]. Over-additive effects of MP-AzeFlu has been observed for certain symptoms, most notably nasal congestion. This may be due to the fact that MP-AzeFlu is more than a simple fixed dose combination product, comprising anti-histaminic, mast-cell stabilizing, anti-leukotriene and anti-inflammatory properties [3]. MP-AzeFlu is well tolerated by patients both in RCTs and in real life, but a bitter taste (due to azelastine) has been reported, and traditionally classified as an adverse event. We hypothesized that MP-AzeFlu may induce some of its beneficial effects via a bitter taste receptor mediated pathway, specifically activation of bitter taste sensing type 2 receptors (TAS2R).

TAS2Rs belong to the family of G-protein coupled receptors (GPCRs) which recognize a wide range of substances. Previously, TAS2Rs were found in the oral cavity, their function being to prevent intake of harmful substances, which often taste bitter [4]. More recent evidence shows that these receptors are present both in the human upper airway mucosa and lower airway smooth muscle [5–8], and have functions independent of taste. Their activation can increase ciliary beat frequency and the release of anti-microbial peptides [4], inhibit histamine and prostaglandin release from human mast cells [7] and induce airway dilation in the lower airways [5, 6].

The present study was designed to explore if MP-AzeFlu has the ability to activate bitter taste receptors without the involvement of the histamine system using an in vitro model of isolated murine airways (known to have a weak histamine receptor system) (for method see Additional file 1). Since no TAS2R antagonists currently exist, proof of TAS2R agonism was inferred by (i) exclusion of other pathways and (ii) by mimicking the effects of a known TAS2R agonist (i.e. chloroquine).

**Fig. 1:**
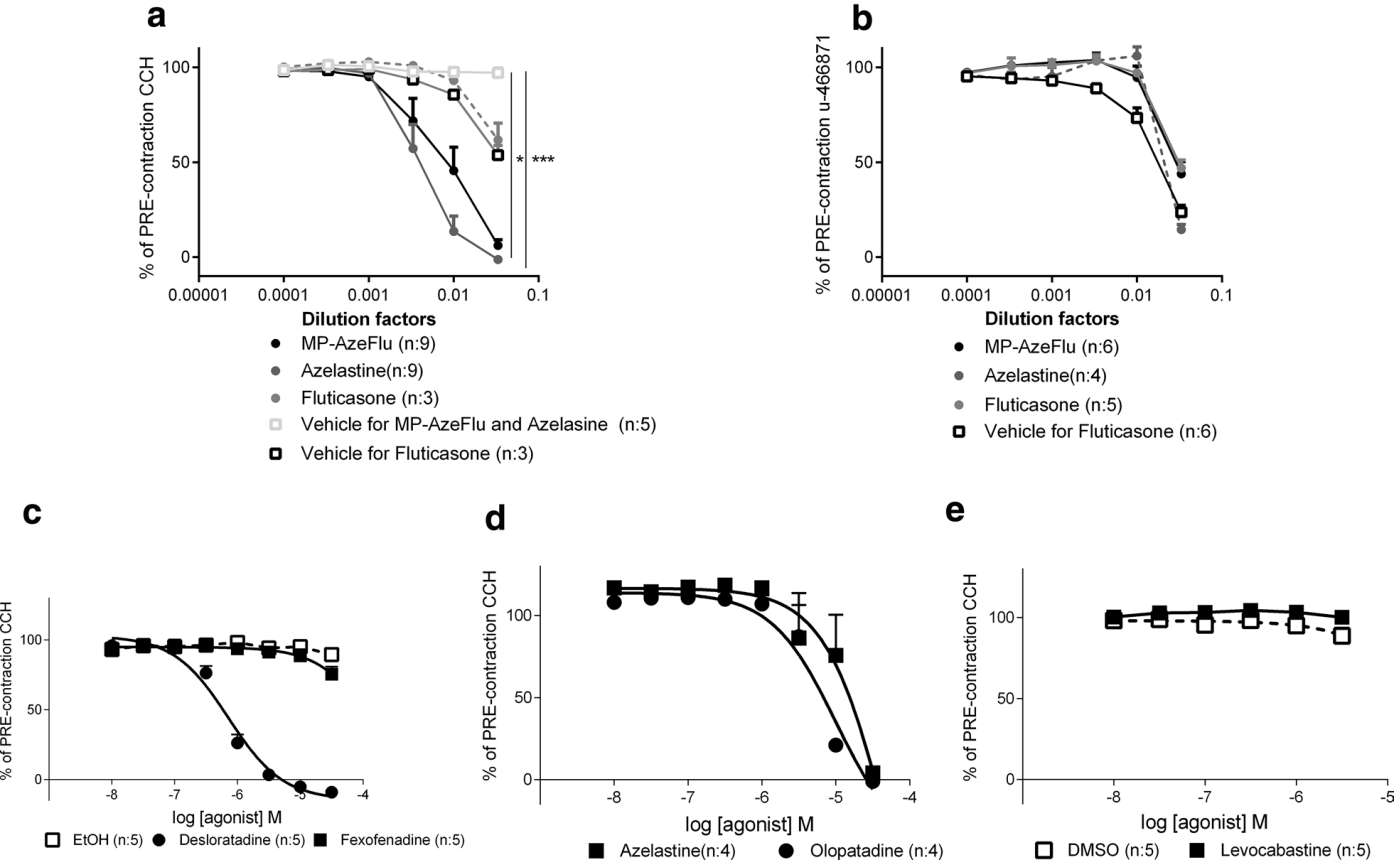
Induced airway dilations on pre-contracted isolated airways. **a** Tracheal segments were pre-contracted with CCH, and increasing concentrations of MP-AzeFlu, azelastine and fluticasone were added. As fluticasone is diluted in DMSO, a separate DMSO control is provided. All other compounds are diluted into KH Buffer. ** MP-AzeFlu vs Fluticasone ***.Azelastin vs Fluticasone. **b** Segments were pre-contracted with U-466871, and increasing concentrations of MP-AzeFlu, azelastine and fluticasone were added. **c** Desloratadine and fexofenadine induced airway dilations. **d** Olopatadine and Azelastine induced airway dilations **e** Levocabastine induced airway dilations. **c**–**e** are divided into three graphs dependent on the different vehicle

The results show that MP-AzeFlu, azelastine but not fluticasone induced a strong relaxation of carbachol (CCh)-induced contractions, and a modest relaxation to the thromboxane receptor agonist U-46619-induced tone of mouse trachea (Fig. 1a, b). The bitter taste receptor TAS2R agonist, chloroquine (acting on the subtypes TAS2R3, TAS2R10 [6]), dilated isolated murine airways in the same dose-dependent manner as MP-AzeFlu and azelastine. The chloroquine induced dilation was more potent when the segments had been pre-contracted with carbachol than with U-46619 [6]. At high concentrations, fluticasone induced a weak relaxation of CCh and U-46619 pre-contracted tissues, but this was likely due to the vehicle used, as vehicle alone induced a similar relaxation.

Though it is well known that mice have a weak histamine receptor system, histamine receptors H1, H2 and H3 were separately pharmacologically inhibited prior to addition of azelastine, to assess the role of this system in relaxation. Chloroquine was used as a comparison and to evaluate the bitter taste effect. The relaxation induced by MP-AzeFlu, azelastine and chloroquine were unaffected by the presence of mepyramine, metiamide and thioperamide, known to block the dilatory activity of the H1, H2 and H3 receptors, respectively (Additional file 2: Fig S1).

The mechanisms behind bitter taste receptor-mediated relaxations are not yet known. Evidence suggests that there is more than one common pathway for all TAS2Rs [6]. In this study, pathways associated with relaxation of the smooth muscle, as well as possible bitter taste transduction pathways were evaluated. Nitric oxide (NO) and carbon monoxide (CO) increase intracellular cGMP and NO activates K+ channels, all of which lead to smooth muscle relaxation. Prostaglandins are also known to mediate relaxation, through increases in intracellular cAMP [5]. Therefore, L-NAME (which blocks NO synthase), zinc protoporphyrin-9 (which inhibits CO synthesis) and indomethacin (to inhibit prostaglandin production) were added to pharmacologically inhibit these pathways. However, none of these agents affected relaxation (Additional file 2: Fig S2).

The relaxation seen from MP-AzeFlu was not a general anti-histamine effect. To verify the possibility of an anti-histamine class effect, the impact of four other antihistamines, (desloratadine, fexofenadine, olopatadine and levocabastine) on CCh-induced pre-contraction were investigated using the same set up used for MP-AzeFlu and azelastine. The relaxatory capacity of azelastine at concentrations comparable to other anti-histamines was additionally assessed. The result precludes a general anti histamine effect, instead strengthening the bitter taste theory. Olopatadine [9] and desloratadine [10], both known for their bitter taste, relaxed airway segments in a way that resembled the effects of MP-AzeFlu and azelastine (Fig. 1c, d). Fexofenadine and levocabastine, which are not associated with a bitter taste, did not induce relaxation (Fig. 1c, e).

The effects induced by bitter taste activation in the airways are, as stated previously, not fully known. A possible candidate for the mediation of the presently postulated effects of bitter taste receptors in allergic rhinitis may be related to epigenetic histone modification. There are at least two levels at which the role of histone modifications is manifested. One is the regulation of cells that contribute to the allergic inflammation (T cells and macrophages) and those that participate in airway remodeling (myo-fibroblasts). The other is the direct association between histone modifications and allergic phenotypes [11]. Hence, it is tempting to speculate that MP-AzeFlu, in addition to its previously well documented anti-allergic effects, also could function as an inhibitor of histone-modifying enzymes, something that might explain its over-additive effects in allergic rhinitis.

The effect that MP-AzeFlu notable had on the smooth muscle could, based on our data, be due to the activation of TAS2R. Activation of these receptors in the nose have been shown to increase ciliary beat frequency and the release of anti-microbial peptides lending to a reduction of nasal congestion and the formation of biofilm [4]. However, TAS2R may also induce relaxation of the smooth muscle on the vascular smooth muscle. Relaxation of vascular smooth muscle in the nasal passages would dilate those vessels, causing an increase in congestion. Here the general anti-histamine properties of MP-AzeFlu may be of importance as anti-histamines can induce a smooth muscle contraction [12]. The use of MP-AzeFlu in the case of AR would then only lead to the beneficial effects of a TAS2R activation.

In summary, MP-AzeFlu is a potent dilator of pre-contracted airways, an effect mediated by the azelastine component. It is clear that this effect is not the result of histamine receptor activation and the study concludes that this is not a general mechanism for the antihistamines, but rather a mechanism specific to bitter anti-histamines. The findings in this work strongly suggests that MP-AzeFlu could activate bitter taste receptor in the same way as chloroquine.

## Supplementary information

**Additional file 1.** Supplementary method.

**Additional file 2: Fig S1.** Airway relaxation induced by MP-AzeFlu (A, D and G), azelastine (B, E and H) and chloroquine (C, F and I) in the presence of histamine antagonists. mepyramine (A-C), metamide (D-F) and thioperamide (G-I), blocking H1, H2 and H3 receptors, respectively. Supplementary. **Fig S2.** Airway relaxation induced by MP-AzeFlu (A, D and G), azelastine (B, E and H)and chloroquine (C, F and I) in the presence of agents blocking NO-production(A-C), prostaglandin activity (D-F) and CO-production(G-I). The CO-blocker were diluted in DMSO; therefore the same amount of DMSO was added in the control experiments.

## Data Availability

The datasets used and/or analysed during the current study are available from the corresponding author on reasonable request.

## Abbreviations

Tas2R: Bitter taste sensing type 2 receptors
AZE: Azelastine hydrochloride
FP: Fluticasone propionate
AR: Allergic rhinitis
CCh: Carbachol
