# Supplementary material for: Effects of MP-AzeFlu enhanced by activation of bitter taste receptor TAS2R
Source: Allergy Asthma Clin Immunol. 2020 Jun 3;16:45. doi: 10.1186/s13223-020-00438-w (PMC7268313; doi:10.1186/s13223-020-00438-w)
Supplement: Supplementary file 1 — Additional file 1. Supplementary method. [file 13223_2020_438_MOESM1_ESM.docx]

**Supplement Method**

**Reagents:**MP-AzeFlu (Dymista®), azelastin, fluticasone, desloratadine, fexofenadine, olopatadine and levocabastine were provided by MEDA (Solna, Sweden). Carbachol, dimethyl sulphoxide (DMSO), U-466871, chloroquine, mepyramine, cimetidine and thioperamide, indomethacin, Nω-Nitro-L-arginine methyl ester hydrochloride (L-NAME) and zinc protoporphyrin-9 were obtained from Sigma (St. Louis, MO, U.S.A). Bradykinin was obtained from Polypeptide (Strasbourg, France). Fungizone and penicillin/streptomycin were obtained from Invivogen (San Diego, CA, USA).

**Tissue preparation:** Male Balb/C mice (Charles River, Germany, 19-25 g) were maintained according to the ethical guidelines described in ethical permissions N258/13 and N153-11. After an acclimatization period of at least one week, mice were sacrificed by cervical dislocation and the lung-heart package were rapidly removed and transferred to ice-cold Krebs-Henseleit (KH) buffer solution (144mM Na^+^, 5.9mM K^+^, 2.5mM Ca^2+^, 1.2mM Mg^2+^ , 131.1mM Cl^−^, 1.2mM H_2_PO_4_^−^, 25mM HCO_3_^−^, 11mM D-glucose and 0.03 mM EDTA). The trachea was then dissected free from surrounding tissue and divided into four equally sized segments.

**In vitro set up:** Mice segments were placed in a tissue myograph (Organ Bath Model 700MO; DMT A/S, Aarhus, Denmark) containing 5 mL baths with KH buffer solution at 37°C, bubbled with carbon gas (5% CO_2_ in O_2_). Changes in the smooth muscle contraction were monitored and isometric force measured (ADInstruments, Hastings, United Kingdom). During an equilibration phase of one hour, the tension of the tissue was set to 0.8 mN. To confirm tissue viability, KCl (60mM) was added. A 30 minute equilibration period followed prior to the start of the pharmacological evaluation

**Pharmacologic evaluation:** Pre-contraction was induced by adding either carbachol (CCh -6,5M) or U-466871(-6,5M). When a stable contraction level was reached, MP-AzeFlu, azelastin or fluticasone and their matched vehicles were cumulatively added. MP-AzeFlu is a mixture of 1 mg/mL azelastine and 0.35 mg/mL fluticasone. Azelastin and fluticasone were therefore prepared separately at these concentrations. Azelastine, fluticasone and MP-AzeFlu were then similarly serially diluted in baths. In follow-up experiments, fluticasone was excluded, as it did not induce relaxation. To investigate a possible effect of histamine receptors in the MP-AzeFlu-induced relaxation, tissues were pre-contracted with carbachol (CCH, -6,5M) followed by desloratadine, fexofenadine, olopatadine, levocabastine, or azelastine and their matched vehicles were cumulatively added. In these experiments, azelastine was diluted in the same manner as the other anti-histamines to ensure a more accurate comparison.

In the rest of the experiments, chloroquine was included as a relaxatory compound, together with MP-AzeFlu and azelastin. Azelastin was diluted to match the azelastin concentration found in MP-AzeFlu. The antagonists mepyramin(1µM), ciametimide(20 µM) and thioperamide(1µM) were used, blocking the H1, H2 and H3 receptors, respectively. Indomethacin (3µM), L-NAME(100µM) and Zinc Protoporphyrin-9(100µM) were used to assess the cAMP and cGMP pathways. The histamine antagonists, indomethacin and L-NAME were all added 15 minutes before the pre-contraction. Zinc Protoporphyrin-9 was added when the pre-contraction level had stabilised. All data are presented as means ± SEMs
