# Supplementary material for: Effects of MP-AzeFlu enhanced by activation of bitter taste receptor TAS2R
Source: Allergy Asthma Clin Immunol. 2020 Jun 3;16:45. doi: 10.1186/s13223-020-00438-w (PMC7268313; doi:10.1186/s13223-020-00438-w)
Supplement: Supplementary file 2 — Additional file 2: Fig S1. Airway relaxation induced by MP-AzeFlu (A, D and G), azelastine (B, E and H) and chloroquine (C, F and I) in the presence of histamine antagonists. mepyramine (A-C), metamide (D-F) and thioperamide (G-I), blocking H1, H2 and H3 receptors, respectively. Supplementary. Fig S2. Airway relaxation induced by MP-AzeFlu (A, D and G), azelastine (B, E and H)and chloroquine (C, F and I) in the presence of agents blocking NO-production(A-C), prostaglandin activity (D-F) and CO-production(G-I). The CO-blocker were diluted in DMSO; therefore the same amount of DMSO was added in the control experiments. [file 13223_2020_438_MOESM2_ESM.docx]

Additional file 2

Supplmentary figures





Supplementary Fig S1. Airway relaxation induced by MP-AzeFlu (A, D and G), azelastine (B, E and H) and chloroquine (C, F and I) in the presence of histamine antagonists. mepyramine (A-C), metamide (D-F) and thioperamide (G-I), blocking H1, H2 and H3 receptors, respectively.





Supplementary Fig S2. Airway relaxation induced by MP-AzeFlu (A, D and G), azelastine (B, E and H)and chloroquine (C, F and I) in the presence of agents blocking NO-production(A-C), prostaglandin activity (D-F) and CO-production(G-I). The CO-blocker were diluted in DMSO; therefore the same amount of DMSO was added in the control experiments.
